# Impacts of forests on children’s diet in rural areas across 27 developing countries

**DOI:** 10.1126/sciadv.aat2853

**Published:** 2018-08-15

**Authors:** Ranaivo A. Rasolofoson, Merlin M. Hanauer, Ari Pappinen, Brendan Fisher, Taylor H. Ricketts

**Affiliations:** 1 Gund Institute for Environment, University of Vermont, 617 Main Street, Burlington, VT 05405, USA.; 2 Rubenstein School of Environment and Natural Resources, University of Vermont, Aiken Center, 81 Carrigan Drive, Burlington, VT 05405, USA.; 3 School of Forest Sciences, University of Eastern Finland, Joensuu Campus, Yliopistokatu 7, 80101 Joensuu, Finland.; 4 School of Business and Economics, Sonoma State University, 1801 East Cotati Avenue, Rohnert Park, CA 94928, USA.; 5 Environmental Program, University of Vermont, Bittersweet, 151 South Prospect Street, Burlington, VT 05405, USA.

## Abstract

Micronutrient deficiency affects about a third of the world’s population. Children in developing countries are particularly vulnerable. Consequences include impaired cognitive and physical development and increased childhood morbidity and mortality. Recent studies suggest that forests help alleviate micronutrient deficiency by increasing dietary diversity. However, evidence is mostly based on weakly designed local case studies of limited relevance to global policies. Furthermore, impacts of forests on diet vary among communities, and understanding this variation can help target actions to enhance impact. We compile data on children’s diets in over 43,000 households across 27 developing countries to examine the impacts of forests on dietary diversity. We use empirical designs that are attentive to assumptions necessary for causal interpretations and that adequately account for confounding factors that could mask or mimic the impact. We find that high exposure to forests causes children to have at least 25% greater dietary diversity compared to lack of exposure, a result comparable to the impacts of some nutrition-sensitive agricultural programs. A closer look at a subset of African countries indicates that impacts are generally higher for less developed communities, but highest with certain access to markets, roads, and education. Our results also indicate that forests could help reduce vitamin A and iron deficiencies. Our study establishes the causal relationship between forests and diet and thus strengthens the evidence for integrating forest conservation and management into nutrition interventions. Our results also suggest that providing households some access to capital can increase the impact of forest-related interventions on nutrition.

## Teaser

Nearby forests improve children’ diets in developing countries, and access to capital enhances the effect.

## INTRODUCTION

Deficiencies in micronutrients, such as vitamins and minerals, are one of the world’s major nutritional challenges, affecting over 2 billion people worldwide (*1*). Often symptomless, and thus unnoticed, they nevertheless have important health consequences such as reduced cognitive and physical development, capacity and productivity, and increased childhood morbidity and mortality. Children are particularly vulnerable, and micronutrient deficiency is more prevalent in developing countries (*2*). Interventions to tackle the problem range from micronutrient supplementation to agricultural programs aimed at combating lack of food and dietary diversity (*2*). The potential role of forests in diversifying diet has led to recent calls for the reassessment of forest conservation and management in nutrition policies and for making forest-related interventions more nutrition-sensitive (*3*–*5*).

**Fig. 1:**
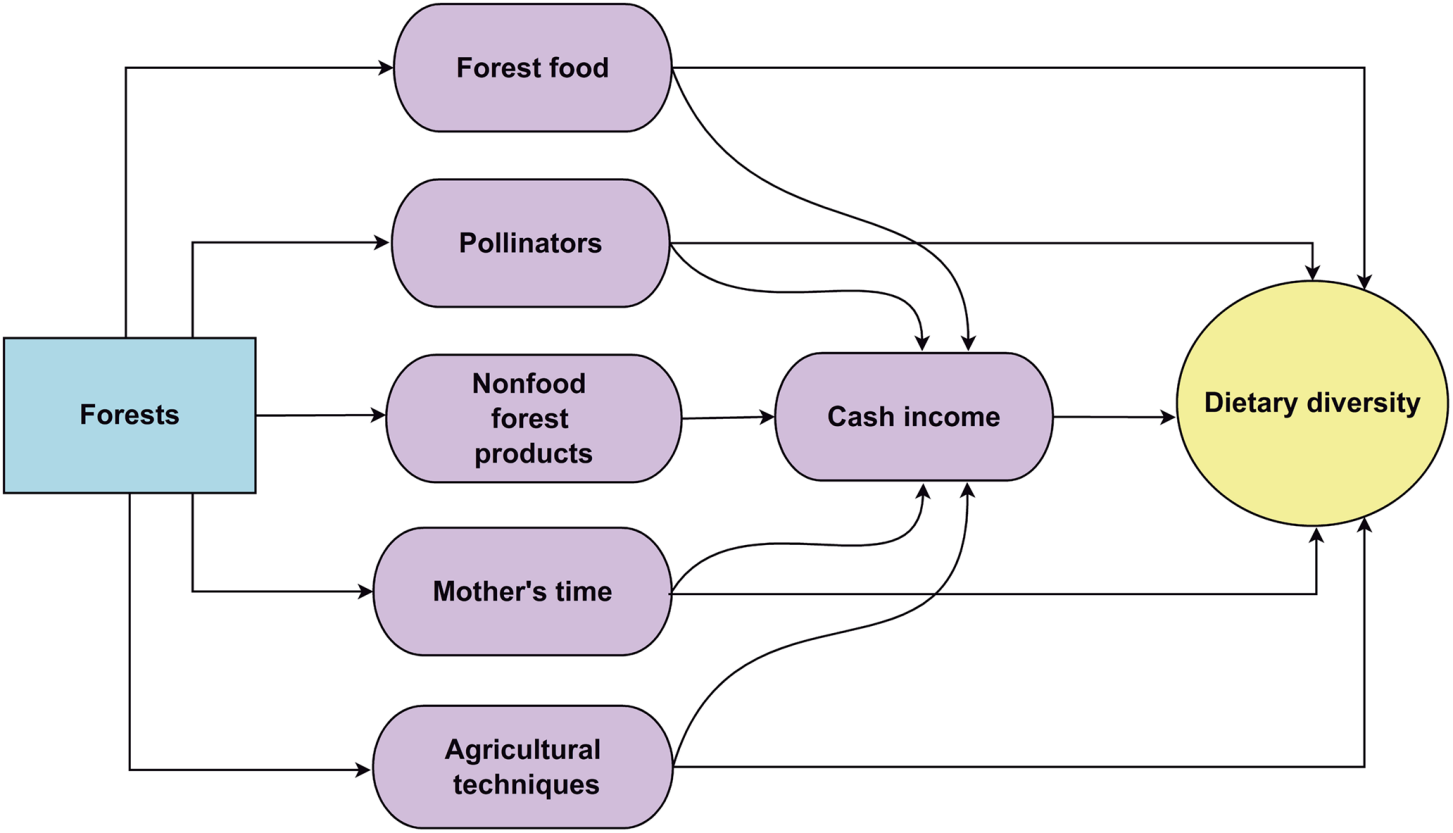
Causal pathways between forests and dietary diversity.

Forests affect dietary diversity through diverse pathways (Fig. 1). Forest food products, including diverse animal, plant, and mushroom species, are commonly collected by rural forest people in developing countries (*5*). While forest foods do not universally form a substantial part of a diet, they supply essential micronutrients and contribute significantly to nutrition in some contexts (*6*). For example, in rural forest-dependent households in Cameroon, forest foods contribute 93% of women’s vitamin A intake, 100% for sodium, 85% for iron, 88% for zinc, and 89% for calcium (*7*). Forests shelter pollinators upon which over 70% of leading global crops depend, representing 35% of the food supply (*8*). Pollination is crucial for the production of fruits and vegetables that are sources of essential micronutrients (*9*) and improve crop quality and quantity. For example, an experiment by Klatt *et al*. (*10*) shows that bee-pollinated strawberries have improved quality and quantity compared to wind-pollinated and self-pollinated strawberries. Access to forest products (timber and nontimber) can affect dietary diversity through increased income and disposable time. A study covering 24 developing countries found that, on average, forest products contribute 22% of total income (subsistence and cash) of rural forest households (*11*). Part of the forest income is sold for cash (*11*), which can be used to buy foods or inputs for agricultural production. Proximity to forest products (for example, firewood) can also affect the time women allocate to nutrition-related activities, such as food preparation or agricultural production (*12*). In Nepal, deforestation was associated with an average increase of 1.13 hours in the time women daily spent to collect fuelwood, leaf fodder, and grass for livestock feed (*13*). Finally, there are also suggestions that agricultural techniques in forested areas often involve diverse crops that may be conducive to a more diverse diet (*14*).

To efficiently integrate forest management into nutrition policies, strong evidence about impacts of forests on diets is needed. However, few studies have explored the relationships between forests and dietary diversity. Further, with the exceptions of the studies of Ickowitz *et al*. (*14*) and Galway *et al*. (*15*), these studies focus on a specific location or country [for example, (*12*, *16*)]. While they generally suggest positive relationships between forests and dietary diversity, the specific spatial focus of these studies renders difficult the applicability of their findings to global policies.

Those existing studies generate evidence based on point estimates (for example, regression coefficients) of the magnitude of the relationships between forests, dietary diversity, and other correlates of dietary diversity, but causal interpretations are difficult. The assumptions necessary for point estimates are strong and not often tested or dealt with in a transparent manner, reducing the credibility of these estimates (*17*, *18*). Credibility can be greatly improved by moving away from a focus on controlling for simple correlates of dietary diversity to a systematic account and control for confounding factors that affect both forest location and dietary diversity. These confounders are the sources of systematic bias in the estimators of impact (*18*). Therefore, existing studies on the relationships between forests and dietary diversity generate important hypotheses that still need to be tested to make clear conclusions about causal impacts.

Heterogeneity of impacts, that is, how different communities or different groups within a community are affected by natural systems (for example, forests), is not well covered in the empirical literature linking natural systems and human health (*19*). The ecological transition theory posits that poorer people rely to a greater extent on natural systems (for example, forests) for the provisioning of services important to health (for example, food) (*19*). Poorer people are therefore more likely to be affected by natural systems. As communities become more developed, they tend to substitute natural capital with other types of capital (for example, physical capitals: roads and markets; human capital: education) for the provisioning of these services.

Therefore, level of development and access to markets, roads, and education are potential variables moderating the impact of forests on dietary diversity. Greater access to markets and roads can reduce dependence on forests and, thus, the impact of forests on diet. Markets provide an alternative source of food. Roads promote access to external food products. Markets and roads can also open opportunities for non–forest-related source of income that is used to buy food. However, markets and roads can also increase the impact of forests on dietary diversity. They can facilitate the exploitation or transformation of forest benefits into more diverse food, including through cash income. Higher levels of education can allow households to better exploit and transform forest benefits into more diverse food, particularly as more educated parents are more diet-conscious and, thus, are more likely to provide higher-quality food to their children (*20*). Understanding these moderating variables could shed light on who is likely to gain or lose from integration of forests and nutrition policies, where to invest in such integration to maximize impacts, and how to enhance impacts of forests on dietary diversity through development actions or actions on access to markets, roads, and education.

**Fig. 2:**
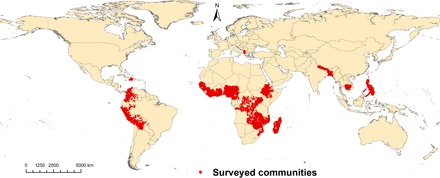
Distribution of communities included in the analyses. The data set includes 43,011 households in 27 developing countries.

We examine the impacts of forests on dietary diversity in rural areas of developing countries. We contribute to the literature in several ways. First, we expand the spatial scope to 27 countries across four continents (Fig. 2). Second, we pay close attention to the assumptions necessary for causal interpretations, so that our empirical designs yield credible estimates of impacts. Finally, we examine how impacts vary as a function of development and access to markets, roads, and education. Through these contributions, our study provides an important step toward strengthening the evidence for integrating forest conservation and management into the list of nutrition interventions.

### Dietary diversity

We measure dietary diversity using the individual dietary diversity score (IDDS) developed by the Food and Agriculture Organization of the United Nations (*21*). IDDS is a simple count of food groups an individual has consumed over a 24-hour period and a standard measure of the quality, particularly micronutrient adequacy, of the diet (*22*). Our IDDS ranges from 0 (the child did not eat any food item of any of the food group) to 10 (the child ate at least 1 food item of each of 10 food groups) (Materials and Methods).

IDDS gives equal weight to each food group. Thus, it may overlook food groups that provide key micronutrients. Given the health consequences of and high number of people affected by vitamin A and iron deficiencies in developing countries (*1*), we look at whether children within a household have consumed vitamin A– or iron-rich food items over a 24-hour period (see Materials and Methods for the definition of vitamin A– and iron-rich food).

### Exposure to forest

Following the United Nations Environment Programme definition of closed forests, we designate as forest an area with a minimum of 40% tree cover (*23*). We define forest households (that is, with high exposure to forest) as those living in communities located within 3 km from forest edges and with at least 30% of the area within 5-km buffer around the community centers covered by forests. Nonforest households (that is, lacking exposure to forest) are those farther than 8 km from forest edges (Materials and Methods).

### Definition of impact

We define impact of high exposure to forest as the difference between the average dietary diversity of forest households and the average counterfactual dietary diversity for these same forest households had there been no nearby forest [formally, the average treatment effect on the treated (ATT; Materials and Methods)]. The former is the observed mean dietary diversity of the forest households, but the latter (the counterfactual) is unobserved. Hence, we must assume that the mean dietary diversity of some of the nonforest households represents the average counterfactual dietary diversity for forest households had they not had access to forests. The credibility of the impact estimate depends on the plausibility of the assumptions we make when identifying this counterfactual. Precise point estimates of impacts often require strong assumptions about counterfactual outcomes, which can diminish their credibility (*17*). We use various estimation strategies with varying levels of assumptions to both bound and calculate point estimates of impacts.

Concerning vitamin A– or iron-rich food consumption, impact of high exposure to forest is the difference between the percentages of forest households where children ate vitamin A– or iron-rich food and the counterfactuals of these percentages had forest households lacked exposure to forest.

## RESULTS

### Estimates of impacts of forests on dietary diversity

We start with a partial identification approach (*24*), which allows us to place the estimate of impact within a credible range without invoking strong and opaque assumptions, including those about the baseline dietary diversity, which is missing as each household was surveyed only once. The mean dietary diversity for forest households across the 27 countries is 3.12. Without making any assumptions, we know that the counterfactual dietary diversity cannot be higher than 10 (the highest possible IDDS) or lower than 0 (the lowest possible IDDS). We thus define the impact estimate to be within a range delimited by a lower bound of 3.12 − 10 = −6.89 and an upper bound of 3.12 − 0 = 3.12.

**Fig. 3:**
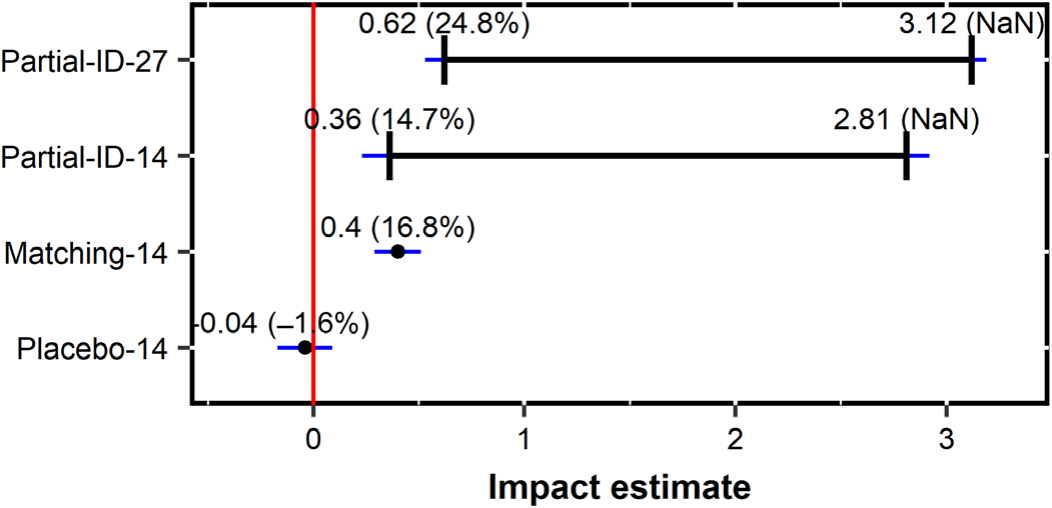
Estimated impacts of forests on dietary diversity. Partial-ID-27, partial identification for 27 countries; Partial-ID-14, partial identification for 14 African countries; Matching-14, matching design for 14 African countries; Placebo-14, placebo test for the matching design for 14 African countries. Values in parentheses, impact expressed in percent of the average dietary diversity of nonforest households. NaN, not a number (undefined). Blue bars, 95% confidence intervals.

To narrow the range of impact estimates, we invoke the weak and plausible assumption (formally, the Monotone Treatment Selection Assumption) that, without forest, forest households would have counterfactual average dietary diversity no higher than the average dietary diversity of the nonforest households (2.50). This assumption is plausible given that remaining forests are often “selected” toward remote and difficult access lands, away from markets and infrastructure, with lower agricultural potential, and where households are more socially disadvantaged than in nonforested areas (*25*)—facts that are generally supported in our data (Table 1). Hence, we move the lower bound from 3.12 − 10 = −6.89 to 3.12 − 2.50 = 0.62. We thus refine the impact estimate to be within the range of 0.62 to 3.12 (Fig. 3).

**Table 1:** Means and SDs (in parentheses) of characteristics of forest and nonforest households across 27 countries.

**Variable**	**Forest household**	**Nonforest household**
IDDS	3.12 (2.29)	2.50 (2.05)
Communities in lands suitable for agriculture (%)	38.22 (48.60)	45.15 (49.76)
Slope (°)	2.66 (3.32)	1.04 (1.48)
Elevation (m)	586.59 (562.83)	627.27 (564.34)
Distance to a road (km)	10.94 (26.09)	2.84 (3.18)
Distance to a city (km)	40.85 (36.34)	32.66 (25.37)
Communities in areas with low livestock density (%)	74.88 (43.37)	19.52 (39.63)
Communities in areas with medium livestock density (%)	18.29 (38.66)	56.77 (49.54)
Communities in areas with high livestock density (%)	6.83 (25.23)	23.71 (42.53)
Community GDP (US$ billion Purchasing Power Parity)	0.87 (1.26)	1.37 (1.34)
Population size (individuals)	6,139.85 (10,561.69)	14,445.98 (35,939.32)
Education of head of household (years)	5.18 (4.02)	3.70 (4.23)
Age of head of household (years)	38.73 (12.76)	39.34 (12.82)
Household size (individuals)	6.32 (2.62)	6.95 (3.69)
Children younger than 5 years (individuals)	2.02 (0.89)	2.22 (1.14)

We also apply similar partial identification approach to a subset of 14 sub-Saharan African countries that are the focus of further analyses and estimate impact for these countries to be within the range of 0.36 to 2.81 (Fig. 3).

To generate a precise point estimate of impact, we use statistical matching. Matching controls for observed confounding variables by selecting nonforest households that are similar to forest households in terms of these variables (see Materials and Methods for more on confounders). After matching, we invoke the strong assumption that the average dietary diversity of the matched nonforest households represents the average counterfactual dietary diversity for forest households [formally, the Conditional Mean Independence Assumption (CMIA; Materials and Methods)]. We restrict the use of matching to 14 sub-Saharan African countries because the CMIA is more likely to be plausible for this subset of countries than for all 27 countries (Materials and Methods). The average difference between the dietary diversity of forest and the matched nonforest households is 0.4 (95% confidence interval, 0.29 to 0.51; Fig. 3).

Further, the CMIA is plausible if there are no unobserved confounders or if they are sufficiently correlated with and thus captured by the observed ones (*18*). We indirectly test this assumption with the placebo test (*26*). We define the matched nonforest households from the primary analysis as placebo households. We then match these placebo households to other nonforest households using the same matching and postmatching procedures. After matching, both the placebo and their matched households have similar observed confounders and both have no forests. Therefore, we should not observe large differences between the dietary diversity of the placebo and their matched households if unobserved confounders are captured by the observed confounders. We detect a small average difference (−0.04) that is statistically insignificant (95% confidence interval, −0.17 to 0.09; Fig. 3), supporting the adequacy of our empirical design.

### Heterogeneity of impacts of forests on dietary diversity

**Fig. 4:**
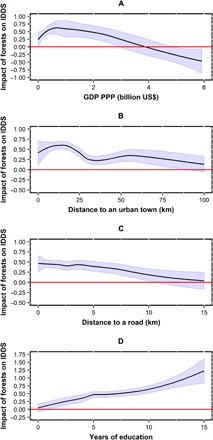
Heterogeneity of impacts of forests on dietary diversity. (**A**) Variation of impact with community GDP. (**B**) Moderating effect of distance to the nearest urban town. (**C**) Moderating effect of distance to the nearest road. (**D**) Moderating effect of the education of the head of household. *Y* axis represents the difference in dietary diversity (IDDS) between forest and similar nonforest households. Blue bands, 95% confidence intervals.

The ecological transition theory (*19*) postulates that poorer communities are more affected by forests than richer communities. To examine this postulate, we assess how impact (difference in dietary diversity between forest and counterfactual households) varies with the gridded gross domestic product (GDP) of the areas where communities are located (gridded GDP, that is, different grids within a country have different GDP values to allow variation within the country; see table S1 for scales of data). GDP is a standard measure of community economic development. Our results suggest that impacts are significantly positive in poor communities (with a peak of about 0.63 at the community GDP of US$0.7 billion), become statistically insignificant between the community GDP of US$3 billion and 5 billion, and then significantly negative above the community GDP of US$5 billion (Fig. 4A).

Further, we estimate the specific moderating effects of access to markets, roads, and education on impacts of forests on dietary diversity. The moderating effects of access to market (proxied by distance to the nearest urban town) and access to road (proxied by distance to the nearest road) have similar trends (Fig. 4, B and C). At low distances, the impact of forests on dietary diversity is significantly positive and then becomes statistically insignificant at larger distances. However, while the effect of forests seems to peak at a distance of 15 km from the nearest town (with a maximum of 0.62), forest effects steadily decrease with increasing distance to the nearest road (with a maximum of around 0.5 at 0 km). The impact of forests on dietary diversity increases markedly with increasing education of heads of households. At low education levels (0 to 1 year), effects are small and statistically insignificant but increase steadily, become significant, and exceed 1.0 at 15 years of education (Fig. 4D).

### Estimates of impacts of forests on vitamin A– and iron-rich food consumption

**Fig. 5:**
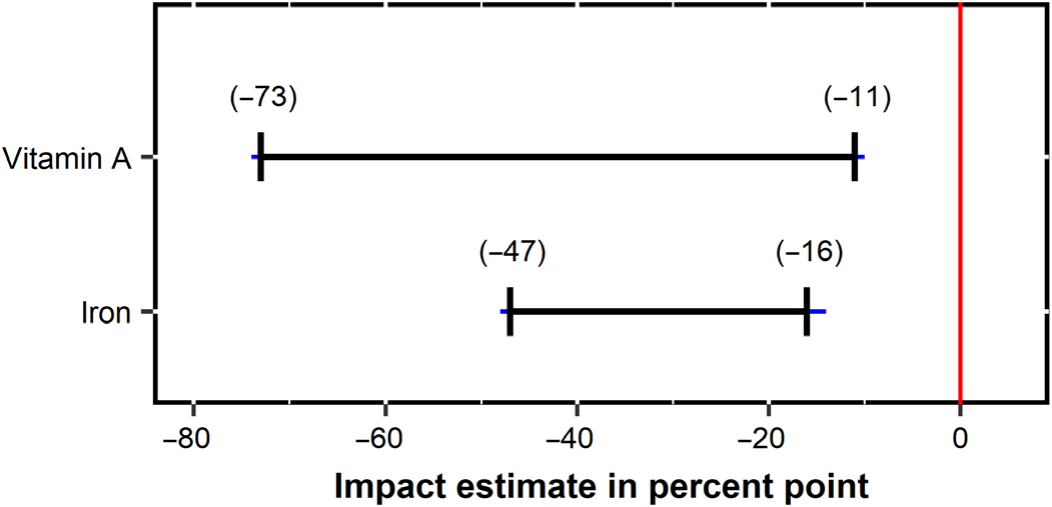
Estimated ranges of impacts of forests on the prevalence of households where children ate vitamin A– or iron-rich food in 27 developing countries. Blue bars, 95% confidence intervals.

Using a partial identification approach (Materials and Methods), we find that high exposure to forest increases the prevalence of households where children ate vitamin A– or iron-rich food by at least (lower-bound estimates) 11 and 16 percent points, respectively (Fig. 5).

## DISCUSSION

Our results indicate that greater exposure to forests, on average, has positive effects on children’s dietary diversity in developing countries. Even our most conservative estimates (that is, the lower bounds of the estimates from partial identification) detect significantly positive impacts. However, while our average estimates of impacts are significantly positive, impacts are heterogeneous across different communities or households. Impacts generally appear positive and greater in communities with lower level of development but seem to be greatest at moderate (not lowest) development levels. Greater access to markets, roads, and education generally improves impacts of forests on dietary diversity.

Our results confirm the generally positive relationship between forests and dietary diversity in the literature (*12*, *14*–*16*). Our results strengthen the credibility of this evidence base because the assumptions embedded with our empirical design are weak, plausible, and transparently examined. When we invoke a strong assumption to generate a point estimate, we still detect significantly positive impact, on average. Our point estimate falls within the range of estimates based on weak and plausible assumptions, and our placebo test yields small and statistically insignificant results, lending support to the point estimate’s credibility.

Comparisons with studies on well-known agricultural interventions aiming to improve micronutrient nutrition put our results into perspective. A study in Mozambique (*27*) suggests that a program on biofortification of orange-fleshed sweet potato added 0.66 to the dietary diversity score of intervention children relative to control children (that is, 20% of the average dietary diversity of control children). In Cambodia, estimates of impacts of a homestead food production program vary between −0.4 and 0.3 (that is, −10 and 7% of the average dietary diversity of control children) (*28*). These studies are not directly comparable to ours because of differences in spatial scale and design among other factors. However, even our lower-bound estimates are comparable to their results, indicating that forest conservation and management could have a role in the portfolio of interventions targeted to fight micronutrient deficiency.

Our results support the ecological transition theory (*19*) and reinforce a large body of the literature (*3*, *4*), showing higher impacts of forests on diet in communities with lower level of development. We found the highest impacts at low, but not the lowest, levels of development (Fig. 4A), perhaps because a certain level of capital is needed to enhance impacts. This interpretation is supported by the specific moderating effects of access to markets and roads (physical capitals) and education level of heads of households (human capital). Lowest levels of capitals (largest distances to a market and road and lowest levels of education) appear to make impacts of forests on diet small and not statistically significant. Higher levels of capitals (nearer to a market and road and higher levels of education) seem to render impacts positive, larger, and statistically significant. It looks though that impacts need a certain distance to a market (an urban town) to be maximized, probably because opportunities for non–forest-related and off-farm sources of income make communities in the immediate vicinity of towns less dependent on forest-related food and income.

Vitamin A deficiency is strongly linked with child mortality via a suite of pathways (*29*), one of which, diarrheal disease, is the second leading cause of child mortality across the globe (*30*). A recent comprehensive review of vitamin A deficiency and child mortality (*29*) states that although the exact mechanism is not fully understood, the effect that adequate intake of vitamin A has on child mortality is most likely related to reduction in the frequency and severity of diarrhea and measles. Given our resultant link between vitamin A and forest cover, interventions aimed at reducing childhood vitamin A deficiency in some of the poorest parts of the world (for example, biofortification, supplementation) might consider the role forests could play in delivering effective outcomes, particularly in places where implementation of these interventions is difficult.

We do not have data on and could not control for household income, an important confounder that can bias our point estimator of impact. We do not use the household wealth variable in our data set as a proxy for income because while the wealth variable is a correlate of dietary diversity, it is likely to be a mechanism through which forests affect diet rather than a confounder. Thus, controlling for the wealth variable would block the impact of forests on diet that occurs through this mechanism (see section S1). Therefore, we use other variables that are commonly used to proxy household income for targeting social programs but could not be affected by forest cover at the time of the survey and thus are unlikely to be mechanisms. The result of the placebo test provides some support that these proxy variables together with the other confounders capture the unobserved income variable.

Our large-scale study is important in describing broad patterns of impacts of forests on dietary diversity. However, interpretation and application of our results to finer-scale contexts should be done with caution. For example, we use the threshold of 40% canopy cover to define forests. While the threshold is recommended for global analysis, it is unlikely to satisfy national or regional definitions of forests (*23*). Finer-scale applications will thus benefit from data more sensitive to finer-scale contexts. Application of the partial identification approach to our data allows us to define ranges of impact estimates that are positive. However, the ranges are still quite wide. Time series data gathered on forest and nonforest households could make ranges of estimates more precise (*31*). As for the point estimate, the placebo test result indirectly supports the ability of our empirical design to capture unobserved sources of bias, but it cannot rule them out. Time series data gathered on forest and nonforest households combined with the matching design we used here could yield stronger evidence by eliminating all time invariant sources of bias (*32*).

Our results demonstrate the potential links between policies targeting forest conservation and nutrition. In particular, so-called “nutrition-sensitive interventions” address the underlying determinants of undernutrition and incorporate specific nutrition goals and actions to achieve them [for example, agriculture, social safety nets (*20*)]. Given that forests improve dietary diversity, which is an underlying determinant of undernutrition (*20*), forest conservation is a potential nutrition-sensitive intervention. By collaborating more closely, conservation and public health scholars and practitioners can better incorporate nutrition goals into forest conservation efforts and design and effectively implement actions to achieve these goals. Our study suggests that a certain level of access to capital such as roads, markets, and education can enhance the impact of forest conservation on dietary diversity and thus help achieve nutrition goals. Other studies point to the need to strengthen community access to physical capitals [for example, transport infrastructure, market, storage facilities (*3*, *33*)], social capital [for example, community associations (*3*)], and human capital [for example, education (*4*)] as a way to optimize forest nutritional benefits. Therefore, a mix of natural, built, social, and human capital is likely to augment nutritional benefits of forests. However, care is needed as some capitals (for example, markets and roads) are also drivers of deforestation (*34*) and can thus imperil forest resources important to nutrition.

To conclude, conventional means to tackle undernutrition have focused on increasing the production of staple food. While increased staple food production addresses the energy needs of a growing global population, it has resulted in the simplification of diets, leading to consumption of less diverse and more nutritionally poor foods (*35*). Consequently, there is an emerging emphasis on making food programs more nutrition-sensitive (*20*). On the basis of rigorous empirical designs, our results indicate that forest conservation and management could be part of nutrition-sensitive programs. Greater exposure to forests has led to more diverse diets in rural children in developing countries, and the magnitude of the impacts is at least comparable to other nutrition-sensitive agricultural interventions. Improving the nutrition sensitivity of forest conservation and management interventions can particularly enhance the nutrition of vulnerable communities. Pairing these interventions with measures that increase communities’ access to capital can further enhance the nutrition benefits of forests. In addition to their roles in supporting livelihoods of local communities and biodiversity, mitigating climate change, and other benefits, we present strong evidence that forests contribute to human health through their roles in micronutrient nutrition.

## MATERIALS AND METHODS

### Study design

#### Dietary diversity

We constructed our dietary diversity measure—IDDS—based on diet information of children younger than 5 years collected through the Demographic and Health Surveys (DHS) program administered by the U.S. Agency for International Development (USAID). The DHS program collected nationally representative data on population demography, health, and nutrition in over 90 countries. We compiled data from DHS across 27 developing countries in Africa, Central and South America, Southeast Asia, and Eastern Europe (Fig. 2) and collected in different years between 2000 and 2013.

To create the outcome variable, IDDS, we followed the food grouping recommended by the Food and Agriculture Organization of the United Nations (*21*) comprising 14 food groups: cereals; vitamin A–rich vegetables and tubers; white roots and tubers; dark green leafy vegetables; other vegetables; vitamin A–rich fruits; other fruits; organ meat; flesh meat; eggs; fish; legumes, nuts, and seeds; milk and milk products; and oils and fats. We reduced the food groups to 10. We combined other vegetables and other fruits into one group because many DHS do not disaggregate these two food groups. Similarly, we grouped organ meat, flesh meat, and fish into one group. We also removed oils and fats, as many DHS do not have information on this food group. For a given food group, we assigned a score of 1 if a child’s diet over the previous 24 hours included at least one food item belonging to that group and 0 otherwise. We then summed these values over all food groups to create the IDDS. We averaged the IDDS of children within a household, as the unit of analysis is a household.

We defined vitamin A– and iron-rich food following the guidelines of the Food and Agriculture Organization of the United Nations (*21*). Vitamin A–rich food items are those in the following food groups: vitamin A–rich vegetables and tubers, dark green leafy vegetables, and vitamin A–rich fruits. Iron-rich food items are those included in the combined organ meat, flesh meat, and fish food group.

#### Exposure to forest

Our forest cover data came from the global MODIS Vegetation Continuous Field products. These products are yearly (2000 to 2010) representations of the Earth’s surface in terms of percent tree cover at 250-m spatial resolution (*36*).

The communities surveyed by DHS (referred as “clusters” in DHS documentation) were georeferenced. We were thus able to integrate the DHS data with the forest cover and the spatial confounder data. We associated each household in the surveys with the forest cover of the year of the survey, except for the 2011–2013 surveys, for which we used the 2010 forest cover. We selected 3 km within forest edges as a criterion to define forest households because, on average, people in rural developing countries walk about 35 min to come to the closest forest to collect forest products (*5*). Using the rule of thumb that a person walks about 5 km in an hour, a 35-min walk is about 3-km distance. In addition, pollinators, which are one mechanism through which forests may affect diet (Fig. 1), generally forage within 3 km of nest sites (*37*, *38*). The other criterion that forest covers at least 30% of the area within 5-km buffer of communities is based on studies suggesting that natural forest habitats need to cover at least 30% of a given area to maintain pollination services (*39*). We used 5-km buffer because the locations of communities in DHS were randomly displaced up to 5 km to protect anonymity of survey respondents. Moreover, because of this displacement, communities located between 3 and 8 km from forest edges could actually be within 3 km of forest edges and thus forest households. Given this uncertainty, we excluded households of communities located between 3 and 8 km from forest edges. We also removed urban communities from our analyses, as forests are mainly located in rural areas. Last, we excluded children under 12 months old, as their diet is dominated by breast milk, particularly in low- and lower middle–income countries (*40*). Our final data set comprised 43,011 households (11,338 forest households and 31,673 nonforest households).

#### Confounding characteristics

We identified both site and household level confounders. Forest cover is a site characteristic and, thus, relevant confounders are site characteristics affecting both forest cover and diet. However, households also self-selected themselves whether to migrate or stay in or out forested areas. Therefore, household characteristics that influence both where households choose to live and diet are also relevant confounders. Here, we presented the rationale for choosing each confounder. Fuller description and data sources of the confounding variables are in table S1.

##### Site characteristics

*Variables related to returns to agriculture*. Confounding site characteristics include site variables related to returns to agriculture because of the major role of agriculture in both forest land conversion (*41*) and food availability worldwide.

Returns to agriculture are higher for lands with higher agricultural potential and more easily accessed. Therefore, we controlled for variables that capture land agriculture potential (agriculture suitability, slope, and elevation) and access (distance to a road or a city). In addition to its effects on returns to agriculture, access also influences the availability of marketed food.

*Livestock production*. Livestock production is one of the major drivers of deforestation, including through expansion of pasture lands (*42*). It contributes to human diet by providing animal-based food. Intensive livestock production can also reduce human dietary diversity by promoting monoculture of crops that can be used for both animal feed and human food (*43*). We used the variable ruminant livestock density to control for livestock production.

*Development*. Development is linked to forest cover, particularly deforestation, through complex pathways (*43*, *44*). Development has also been linked to health-related outcomes, including nutrition. Low- and middle-income countries in sub-Saharan Africa and South Asia have higher prevalence of childhood undernutrition than any other regions on the globe, with 42% of children younger than 5 years in East Africa being stunted (*2*). To control for development, we used GDP of the areas where communities are located. The community GDP was converted to U.S. dollars using purchasing power parity factor to adjust for spatial and temporal variations across communities.

*Population*. Larger population triggers more deforestation by putting more pressure on forest resources (*44*). Increasing population size can render an area more attractive to market and thus provide access to marketed food (*45*). We controlled for population size.

##### Household characteristics

*Education*. Level of education affects one’s decision to migrate (*46*). Parents’ education also influences children’s nutrition (*20*). Thus, we controlled for the levels of education of heads of households.

*Proxies for income*. Income or wealth is suggested to determine migration (*47*). Greater income is also associated with improved nutrition (*48*). The DHS data do not have information on household income. We controlled for a combination of variables that are among those widely used to proxy household income for targeting social programs, namely, education and age of heads of households, household size, and number of children younger than 5 years in a household (*49*).

### Statistical analysis

#### ATT, CMIA, and matching

The potential outcome model of causal inference (*50*) posits that each household in our population has two potential dietary diversity outcomes: the dietary diversity under high exposure to forest, *Y*_*i*_(1), and the dietary diversity under lack of exposure to forest, *Y*_*i*_(0). The causal effect of high exposure to forest for a particular household can be defined as the difference between these two potential outcomes

$$\delta_{i}=Y_{i}(1)-Y_{i}(0)$$ (1)

The challenge is that, for a household *i* at a given moment, we can observe either *Y*_*i*_(1) or *Y*_*i*_(0) but not both. The unobserved potential outcome is called counterfactual outcome.

At the population level, we can define the ATT (here, treatment is high exposure to forest)

$$\begin{array}{cc} \text{ATT} & =E[Y_{i}(1)-Y_{i}(0)|D_{i}=1]\\ & =E[Y_{i}(1)|D_{i}=1]-E[Y_{i}(0)|D_{i}=1] \end{array}$$ (2)

where *E*[.] is the expectation operator from probability theory and |*D*_*i*_ = 1 means conditional on the household being under high exposure to forest (that is, being forest household). In other words, the ATT is the difference between the expected dietary diversity of forest households under high exposure to forest, *E*[*Y*_*i*_(1)|*D*_*i*_ = 1], and the expected dietary diversity of these same forest households were they under lack of exposure to forest, *E*[*Y*_*i*_(0)|*D*_*i*_ = 1]. The former, which is the average dietary diversity of forest households, is observed. The latter, which is the dietary diversity of forest households, had they not been exposed to forest, is unobserved (the counterfactual).

The CMIA postulates that, conditional on comparable observed confounders, *X*, between forest and nonforest households, the expected dietary diversity of the nonforest households under lack of exposure to forest, *E*[*Y*_*i*_(0)|*D*_*i*_ = 0], represents the unobserved counterfactual average dietary diversity, *E*[*Y*_*i*_(0)|*D*_*i*_ = 1]

$$E[Y_{i}(0)|D_{i}=1,X]=E[Y_{i}(0)|D_{i}=0,X]=E[Y_{i}(0)|X]$$ (3)

In other words, the expected dietary diversity under lack of exposure to forest, *E*[*Y*_*i*_(0)], for households with similar characteristics is independent of the causal states, that is, whether the households are under high (*D*_*i*_ = 1) or lack of (*D*_*i*_ = 0) exposure to forest. Matching seeks to satisfy the CMIA by processing the data such that the resulting postmatching set of data achieves balance across key confounders, *X*, between the forest and nonforest households. In principle, if outcomes (IDDS) and exposure to forest are determined by observable confounders (*X*), then postmatching balance across *X* allows for identification of the ATT via difference in means between forest and nonforest households. We restricted the use of matching to 14 sub-Saharan African countries where we achieved a reasonable level of postmatching balance on the observed confounders (table S2). Equally important, we adjusted for the remaining postmatching imbalance with the postmatching linear regression bias correction of Abadie and Imbens (*51*). We performed one-to-one matching with replacement using the multivariate Mahalanobis distance measure. We executed exact matching on country. We used the R “matching” package (*52*).

#### Heterogeneity of impacts

To examine the variation of impact with community GDP, we plotted impact (forest household IDDS − counterfactual IDDS) against community GDP using a nonparametric locally weighted scatter plot smoothing (LOESS). LOESS enabled us to analyze the relationship between impact and community GDP without controlling for other variables that correlated with community GDP. We allowed other variables to change with community GDP, as we are interested in how poor and rich communities fare rather than the isolated effect of the community GDP variable.

Distance to an urban town (with at least 5000 inhabitants), distance to a road, and education level of heads of households are potential moderators of impacts. We are therefore interested in their isolated moderating effects, net of the effects of the other variables. To isolate their moderating effects, we used a semiparametric partial linear differencing model (PLM) (*53*). PLM linearly controls for other confounders before calling LOESS to estimate impact as a function of the moderators. Hence, PLM estimates the impact of forests on dietary diversity as a function of the mediators while holding constant the other confounders.

#### Partial identification of impacts of forests on vitamin A– and iron-rich food consumption

The percentages of forest households where all children ate vitamin A– or iron-rich food are 73 and 47%, respectively. Without making any assumptions, the counterfactual percentages must be between 100% (all children of all households ate vitamin A– or iron-rich food) and 0% (all households have children who did not eat any vitamin A– or iron-rich food). Therefore, we bounded the impact estimate to be between 73 − 100 = −27% and 73 − 0 = 73% for vitamin A, and between 47 − 100 = −53% and 47 − 0 = 47% for iron.

Then, we invoked the same Monotone Treatment Selection Assumption as we invoked in the analysis for dietary diversity (see Results). That is, without forests, the counterfactual percentages of households where all children ate vitamin A– or iron-rich food for forest households would be no higher than the percentages for nonforest households (62% for vitamin A and 31% for iron). Thus, the lower bounds of the ranges of impact estimates are reduced to 11% (73 to 62) for vitamin A and 16% (47 to 31) for iron (Fig. 5).

#### Confidence intervals

We computed cluster-robust confidence intervals for all our estimated impacts (including the heterogeneous impacts) with SEs clustered at the community level to account for the nonindependence of households within a community. The confidence intervals of the ranges of estimates from the partial identification approach are based on the procedure described in King and Zeng (*54*). We used the R “clusterSEs” package (*55*) for the confidence intervals of the average impact estimates and the R function developed by Hanauer and Canavire-Bacarreza (*56*) for those of the heterogeneity of impact analyses.

## Supplementary Material

http://advances.sciencemag.org/cgi/content/full/4/8/eaat2853/DC1

## SUPPLEMENTARY MATERIALS

Supplementary material for this article is available at http://advances.sciencemag.org/cgi/content/full/4/8/eaat2853/DC1 Section S1. Why not controlling for the DHS wealth variable? Table S1. Description and sources of the confounding variables. Table S2. Covariate balance between forest and nonforest households in 14 sub-Saharan countries. Reference (*57*)
