# Supplementary material for: Impacts of forests on children’s diet in rural areas across 27 developing countries
Source: Sci Adv. 2018 Aug 15;4(8):eaat2853. doi: 10.1126/sciadv.aat2853 (PMC6093622; doi:10.1126/sciadv.aat2853)
Supplement: http://advances.sciencemag.org/cgi/content/full/4/8/eaat2853/DC1 [file supp_4_8_eaat2853__index.html]

Science Advances | Science Advances

## Supplementary Materials

**This PDF file includes:**

- Section S1. Why not controlling for the DHS wealth variable?
- Table S1. Description and sources of the confounding variables.
- Table S2. Covariate balance between forest and nonforest households in 14 sub-Saharan countries.
- Reference (*57*)

Download PDF

**Files in this Data Supplement:**

- Adobe PDF - aat2853\_SM.pdf
