# Supplementary material for: Impacts of forests on children’s diet in rural areas across 27 developing countries
Source: Sci Adv. 2018 Aug 15;4(8):eaat2853. doi: 10.1126/sciadv.aat2853 (PMC6093622; doi:10.1126/sciadv.aat2853)
Supplement: http://advances.sciencemag.org/cgi/content/full/4/8/eaat2853/DC1 [file aat2853_SM.pdf]

Science Advances
AAAS

advances.sciencemag.org/cgi/content/full/4/8/eaat2853/DC1

Supplementary Materials for

Impacts of forests on children's diet in rural areas across 27 developing countries

Ranaivo A. Rasolofoson*, Merlin M. Hanauer, Ari Pappinen, Brendan Fisher, Taylor H. Ricketts

*Corresponding author. Email: rrasolof@gmail.com

Published 15 August 2018, *Sci. Adv.* **4**, eaat2853 (2018)
DOI: 10.1126/sciadv.aat2853

This PDF file includes:

Section S1. Why not controlling for the DHS wealth variable?
Table S1. Description and sources of the confounding variables.
Table S2. Covariate balance between forest and nonforest households in 14 sub-Saharan countries.
Reference (*57*)

Supplementary Materials

Section S1. Why not controlling for the DHS wealth variable?

The DHS data have a household' s wealth variable constructed from a combination of household assets and housing characteristics. This variable would be a natural proxy for income as a confounder. However, the wealth data were collected in the same years as the forest cover and the diet data. Therefore, forests could affect the wealth variable, which in its turn could affect diet. In other words, the wealth indicated by the variable could be a mechanism through which forests affect diet. Controlling for this variable could block the effects of forests on diet that occur through this mechanism and thus bias the estimator.

To test whether the wealth indicated by the wealth variable is a potential mechanism, we first examine the impact of forests on wealth. If forests have no impact on the wealth variable, we will have some assurance that the wealth variable is not a mechanism and we should use it as a proxy for income among the confounders. We use the same matching design as we use in the investigation of the impact of forests on diet in the main text. The result suggests that households with high exposure to forests have wealth score 0.24 point (95% confidence interval = [0.21, 0.26]) greater than those that lack exposure. Therefore, the wealth variable could be a mechanism. Assuming that greater wealth yields greater diet diversity, controlling for the wealth variable would block the positive impact of forests on diet diversity going through the positive impact of forests on wealth. We thus predict that the estimate of impacts of forests on dietary diversity would be likely to be underestimated if we controlled for the wealth variable.

To test this prediction, we estimate the impact of forests on dietary diversity controlling for the DHS wealth variable. The impact estimate is 0.22 (95% confidence interval = [0.11, 0.33]), which is likely to be underestimated as predicted because it lays below the lower bound of the range of estimates defined in the partial identification approach (Figure. 3 in the main text). Therefore, the DHS wealth variable is likely to be a mechanism and we thus do not control for it. Instead, we used a combination of variables that are among those widely used to proxy household income for targeting social programs, but could not be affected by forest cover at the time of the survey. These variables include education and age of heads of households, household size, and number of children under 5 in a household (*49*).

Table S1. Description and sources of the confounding variables.

**Confounding variable**	**Description**	**Source**
Suitability for agriculture	Suitability of areas for agriculture at community 10 km grid based on climate, soil, and slope constraints. Lands suitable for agriculture comprise those with no, very few, few, and partial constraints. Areas unsuitable for agriculture comprise lands with frequent and very frequent severe constraints, areas with severe climate constraints, lands unsuitable for agriculture, undefined areas (sea), and water.	Global Agro-Ecological Zones – 2000 – Plate 28 (Food and Agriculture Organization of the United Nations, Rome, Italy, International Institute for Applied Systems Analysis, Laxenburg, Austria, 2002; https://webarchive.iiasa.ac.at/Research/LUC/GAEZ/index.htm).
Slope	Average slope in degrees within 5 km buffer around community point	SRTM 90m Digital Elevation Database v4.1 (Consortium for Spatial Information of the Consultative Group for International Agricultural Research, Washington, DC, 2008; http://www.cgiar-csi.org/data/srtm-90m-digital-elevation-database-v4-1).
Elevation	Average elevation in meters within	SRTM 90m Digital Elevation Database

	5 km buffer around community point	v4.1 (Consortium for Spatial Information of the Consultative Group for International Agricultural Research, Washington, DC, 2008; http://www.cgiar-csi.org/data/srtm-90m-digital-elevation-database-v4-1).
Distance to a road	Distance between the community point and the nearest road in meters. The date of the roads in the data ranges from the 1980s to 2010 depending on the country.	Global Roads Open Access Data Set, Version 1 (gROADSv1) (Center for International Earth Science Information Network, Columbia University, New York, Information Technology Outreach Services, University of Georgia, Athens, Georgia, 2013; http://dx.doi.org/10.7927/H4VD6WCT).
Distance to a city	Distance between the community point and the nearest town with at least 5,000 inhabitants in meters, circa 2000	Millennium Ecosystem Assessment: MA Population (Millennium Ecosystem Assessment, NASA Socioeconomic Data and Applications Center, Palisades, New York, 2005; http://dx.doi.org/10.7927/H4CF9N1K).
Ruminant livestock density	Ruminant livestock density at community 10 km grid in Tropical Livestock Unit (TLU) per km^2,	Global Agro-ecological Zones (GAEZ v3.0) (Food and Agriculture Organization of the United Nations, Rome, Italy,

	circa 2000. Based on the information on the carrying capacities of different agro-ecological zones in the study of Pica-Ciamarra, Otte and Chilonda (*57*), we grouped the densities in three categories; Low: 0 to 10 TLU/km^2 (density below any carrying capacity), Medium: 10 to 50 TLU/km^2 (carrying capacities of different agro-ecological zones), High: more than 50 TLU/km^2 (density above any carrying capacity)	International Institute for Applied Systems Analysis, Laxenburg, Austria, 2010; http://gaez.fao.org/Main.html#).
GDP	GDP at community 100 km grid in 1995 USD purchasing power parity: GDP in 2000 for 2000 – 2004 surveys and GDP in 2005 for 2005 – 2013 surveys	W. D. Nordhaus, X. Chen, Global Gridded Geographically Based Economic Data (G-Econ), Version 4 (NASA Socioeconomic Data and Applications Center, Palisades, New York, 2016; http://doi.org/10.7927/H42V2D1C).
Population size	Population count within 5 km buffer around community point: population in 2000 for 2000 – 2004	Gridded Population of the World, Version 4 (GPWv4): Population count adjusted to match 2015 revision of UN

	surveys, population in 2005 for 2005 – 2009 surveys, and population in 2010 for 2010 – 2013 surveys	WPP country totals (Center for Intenational Earth Science Information Network, Columbia University, New York, 2016; http://dx.doi.org/10.7927/H4SF2T42).
Education of head of household	Number of years of education of head of household	Demographic and Health Surveys
Age of head of household	Age of head of household	Demographic and Health Surveys
Household size	Number of members of household that usually live in the household	Demographic and Health Surveys
Number of children under 5 in household	Number of children resident in the household and aged five or under	Demographic and Health Surveys

Table S2. Covariate balance between forest and nonforest households in 14 sub-Saharan countries.

Variable	Mean forest household	Mean non-forest household	Difference of means	Mean raw eQQ difference*
Suitability for agriculture (%)				
- Unmatched	38.63	46.84	-8.21	8.21
- Matched	38.63	37.44	1.19	1.18
Slope (degree)				
- Unmatched	2.28	1.09	1.19	1.19
- Matched	2.28	1.54	0.74	0.74
Elevation (m)				
- Unmatched	756.24	701.27	54.97	68.32
- Matched	756.24	672.87	83.37	84.37
Distance to a road (km)				
- Unmatched	3.89	2.96	0.93	0.93
- Matched	3.89	2.99	0.90	0.91
Distance to a city (km)				
- Unmatched	30.61	35.10	-4.49	5.66
- Matched	30.61	30.08	0.53	1.80
Medium livestock density (%)				
- Unmatched	20.94	56.79	-35.85	35.85
- Matched	20.94	30.38	-9.44	9.48

High livestock density (%)				
- Unmatched	3.97	23.22	-19.25	19.25
- Matched	3.97	3.33	0.64	0.64
GDP (billion US$ PPP)				
- Unmatched	1.15	1.28	-0.13	0.31
- Matched	1.15	0.91	0.24	0.25
Population size (individuals)				
- Unmatched	8516.90	13471.00	-4954.10	5214.80
- Matched	8516.90	8195.50	321.40	848.11
Education of head of household (years)				
- Unmatched	5.16	3.82	1.34	1.40
- Matched	5.16	4.46	0.70	0.70
Age of head of household (years)				
- Unmatched	39.18	38.39	0.79	1.06
- Matched	39.18	37.93	1.25	1.60
Household size (individuals)				
- Unmatched	6.50	6.61	-0.11	0.15
- Matched	6.50	6.34	0.16	0.20
Children under 5 (individuals)				
- Unmatched	2.07	2.16	-0.09	0.10
- Matched	2.07	2.05	0.02	0.09

[*]The mean difference in raw eQQ is a descriptive statistic based on the empirical Quantile-Quantile (eQQ) plot (*52*). It measures the mean distance observed in the eQQ plot when the distribution of a variable is plotted for two different samples, such as forest and non-forest households.
